# Improving engineering students’ understanding of classical physics through visuo-haptic simulations

**DOI:** 10.3389/frobt.2024.1305615

**Published:** 2024-03-21

**Authors:** Guillermo González-Mena, Octavio Lozada-Flores, Dione Murrieta Caballero, Julieta Noguez, David Escobar-Castillejos

**Affiliations:** ^1^ Facultad de Ingeniería, Universidad Panamericana, Ciudad de México, Mexico; ^2^ Servicio Nacional de Bachillerato en Línea–Prepa en Línea, Dirección de Servicios Académicos y Diseño Curricular, Ciudad de México, Mexico; ^3^ Tecnologico de Monterrey, School of Engineering and Science, Ciudad de México, Mexico

**Keywords:** classical physics, visuo-haptic simulators, educational innovation, higher education, science education

## Abstract

**Introduction:** The teaching process plays a crucial role in the training of professionals. Traditional classroom-based teaching methods, while foundational, often struggle to effectively motivate students. The integration of interactive learning experiences, such as visuo-haptic simulators, presents an opportunity to enhance both student engagement and comprehension.

**Methods:** In this study, three simulators were developed to explore the impact of visuo-haptic simulations on engineering students’ engagement and their perceptions of learning basic physics concepts. The study used an adapted end-user computing satisfaction questionnaire to assess students’ experiences and perceptions of the simulators’ usability and its utility in learning.

**Results:** Feedback from participants suggests a positive reception towards the use of visuo-haptic simulators, highlighting their usefulness in improving the understanding of complex physics principles.

**Discussion:** Results suggest that incorporating visuo-haptic simulations into educational contexts may offer significant benefits, particularly in STEM courses, where traditional methods may be limited. The positive responses from participants underscore the potential of computer simulations to innovate pedagogical strategies. Future research will focus on assessing the effectiveness of these simulators in enhancing students’ learning and understanding of these concepts in higher-education physics courses.

## 1 Introduction

The field of education is currently experiencing a significant transformation known as Education 4.0, aligned with the ongoing Industrial Revolution World Economic Forum (2020). The current trend in education emphasizes a shift from traditional, teacher-centered approaches towards more customized, learner-driven practices (UNESCO, 2023). Simultaneously, technological advancements have shown the necessity for educational approaches that not only enrich learning experiences but also develop a deeper comprehension of complex concepts.

Modern technologies, especially those incorporating haptic feedback and embodied learning, are central to the aforementioned revolution. By integrating multi-modal experiences, these technologies have the potential to significantly enhance educational environments, thereby improving the learning process’s overall quality. According to McLinden et al., the presence of receptors spread throughout our skin and the rest of our bodies makes the sense of touch an effective tool for gathering information Mclinden et al. (2019). When engaging in active touch, a dynamic interaction exists between our kinesthetic system and the surrounding environment. This interaction fulfills three fundamental functions: a) acquiring information about the environment; b) recognizing textures, rigidity, and contours; and c) recognizing related characteristics of objects, such as shape and weight Lederman and Klatzky (2009).

While studies have explored the potential of multi-modal technologies in education, there remains a gap in understanding the full impact of these technologies on students’ cognitive processes and learning experiences (Neri et al., 2015; Shaikh et al., 2017; Yuksel et al., 2017; Neri et al., 2018; Neri et al., 2020; Walsh et al., 2020; Walsh and Magana, 2023). This underlines the importance of further research into how visuo-haptic simulations can be optimized during their development to support and implement effective learning strategies.

This work presents the design and development of three visuo-haptic simulators aimed at teaching classical physics concepts. Through interactive engagement, these simulators allow students to explore and physically experience forces, enhancing their learning experience. The objective of this study is to evaluate the usability of these simulators and understand students’ perceptions of their educational value. To this end, the following research questions were proposed:

1. How does visuo-haptic technology influence students’ engagement and perception of learning fundamental physics concepts?
2. What are students’ perceptions regarding the intuitiveness and educational potential of visuo-haptic simulators?
3. What useful information can be derived from students’ interactions and feedback when engaging with these simulations?

To address these questions, the end-user computer satisfaction survey, proposed by Doll and Torkzadeh (1988), was adapted to assess the usability and perceived educational effectiveness of the simulators. This adaptation aims not only to assist educators in designing multi-modal learning scenarios but also to establish a framework for evaluating the impact of these technologies on the learning experience.

This article is structured as follows: The related work is presented in Section 2. Section 3 provides the materials and methods employed in the study. Sections 4 and 5 present the results and discussions related to the design and implementation of the simulators, according to the feedback and outcomes of the questionnaire, respectively. Finally, Section 6 outlines the study’s conclusions, key observations, and potential directions for future research.

## 2 Related work

Classical physics refers to the theories and principles that describe the physical universe’s various phenomena in terms of a handful of fundamental laws and concepts. These classical concepts form a foundational framework that effectively explains a wide range of macroscopic events and phenomena society observes daily. Due to the nature of physics as a fact-based science, students need to interact in some way with the natural phenomena they wish to describe. However, analyzing these phenomena in the classroom is not always feasible.

Hence, the addition of multi-modal interactions has allowed the development of enhanced teaching and learning models. The idea of embodied cognition plays an essential role in this progress. It is a paradigm that considers all forms of human knowledge and cognition “embodied”, as they are acquired through bodily experiences (Mahon and Caramazza, 2008; Maggio et al., 2022). Recent advancements in e-learning courses and Technology Enhanced Learning (TEL) methodologies have experienced an increase in recent years within the fields of Science, Technology, Engineering, and Mathematics (STEM), serving as additional resources to traditional classroom lectures, and they aid students in developing their problem-solving abilities and critical and creative thinking (Park et al., 2010).

According to Taljaard, multi-modal technologies are rapidly gaining attention as effective and novel methods for improving educational practices Taljaard (2016). As a result, there has been a significant surge in the development of educational simulators and learning platforms centered on physics concepts. The Coriolis effect is the way a moving object seems to deviate when seen from a rotating point of reference. Hamza-Lup and Page created a visuo-haptic simulation to explain this effect Hamza-Lup and Page (2012). The simulator allowed participants to manipulate a ball from a rotating surface. Furthermore, the simulation offered participants a synchronized perspective of the rotational movement following the surface. Despite encountering difficulties in navigating the ball during their initial interactions, the majority of participants later perceived the controls to be intuitive. After becoming accustomed to the simulation, the feedback provided by the students was predominantly positive, with a notable 94% expressing enthusiasm for the positive aspects of the simulation.

Zhuoluo et al. performed a study to evaluate the potential of haptic technology for self-learning (Zhuoluo et al., 2019). The authors designed an educational application integrated with a haptic device to teach the concept of friction. The haptic feedback was provided using a 2-degree-of-freedom pantograph device named “Haply”. To ensure seamless communication with the Haply devices, the Unity3D game engine and the Haply application programming interface were used in the development of the visuo-haptic simulation ecosystem. Participants were split into two groups: a control group and an experimental group. Post-experiment evaluations encompassed both a test and a feedback questionnaire. Results showed that the integration of the haptic device not only enhanced the effectiveness of the application but also stimulated increased levels of enthusiasm and motivation among the students.

Yuksel et al.‘s study on friction forces used haptic feedback and visual cues to create the educational visuo-haptic simulation (Yuksel et al., 2019). The simulator simulates the dynamics of frictional forces between two rigid bodies, specifically focusing on phenomena such as stick-to-slip transitions and steady sliding. The study analyzed the impact of variables like object mass, size, and the contacting surface (i.e., friction coefficient) on the nature of the frictional force. Students who had previously enrolled in at least one physics course tested the visuo-haptic simulator. Results highlighted that the participants demonstrated an improved understanding of friction concepts. The authors suggested that improved comprehension resulted from a seamless integration of visual and kinesthetic techniques.

Qi et al. built a visuo-haptic physics simulator to teach the effect of buoyancy (Qi et al., 2020). Buoyancy refers to the upward force exerted on an object submerged in a fluid. The study focused on how visual and haptic feedback could enhance participants’ comprehension of this phenomenon. A 2 x 2 between-subjects design study was conducted. The experiment had four conditions based on the presence or absence of haptic and visual feedback, and participants were randomly allocated to one of these conditions. After an initial evaluation to measure participants’ knowledge of buoyancy, they proceeded to use the simulator and subsequently answered a post-interaction questionnaire. Results suggested that participants in the combined haptic and visual condition exhibited notable progress in their learning outcomes.

In the work of Hamza-Lup and Goldbach, the authors developed a visuo-haptic training tool designed as a gamified scenario (Hamza-Lup and Goldbach, 2021). It was designed to facilitate students’ comprehension of abstract physics concepts, particularly those related to electromagnetism and the fundamental Lorentz force. When a charged particle travels through electric and magnetic fields, this force describes the interaction it encounters. In this study, undergraduate students with non-physics backgrounds volunteered, and they were placed into three groups: the control group, the visuo-haptic group, and the visual group. The control group used only traditional teaching methods. In contrast, the visuo-haptic group used the simulator with haptic feedback features, while the visual group engaged with the simulator without this kinesthetic component. The study’s results showed that the students in the visuo-haptic group not only did better on tests but also showed how advanced visual-haptic interfaces might be able to keep students interested and help them learn more effectively.

A comprehensive review by Crandall and Karadogan emphasizes the pedagogical effectiveness of haptic systems in learning, exploring how these technologies can be designed to align with cognitive theories like Cognitive Load and Embodied Cognition (Crandall and Karadoğan, 2021). This study explores the subtle influence of haptic design on learning effectiveness and suggests optimal strategies for creating haptic simulations. Moreover, a study by DeCoito underscores the transformative potential of digital media in conveying the dynamic and historical development of science, presenting it as a product of its sociocultural context DeCoito (2020). DeCoito showed that using explicit-reflective teaching along with digital tools like scientific timelines and video games helped students understand nature of science concepts better, such as how it is tentative, durable, and self-correcting, and how creativity and sociocultural factors affect scientific inquiry.

The authors’ earlier research mostly looked at the technical progress and immediate educational effects of visuo-haptic simulators (Neri et al., 2015; Shaikh et al., 2017; Neri et al., 2018; Neri et al., 2020). These works have laid a foundational understanding of how kinesthetic and visual feedback can synergize to improve learners’ grasp of complex physics concepts. Our research builds upon these studies, not only exploring the technical and pedagogical efficacy of visuo-haptic simulators but also extending the investigation into user satisfaction and its role in the effectiveness of learning tools. This approach aligns with the emerging recognition within the field of educational technology that learner engagement and satisfaction are critical components of successful educational experiences.

Furthermore, this study employs an enhanced survey methodology to measure user satisfaction with multi-modal applications. This survey could provide researchers with a better understanding of the qualitative aspects of learning with visuo-haptic simulators. This focus on user experience and satisfaction proposes that the effectiveness of educational simulators extends beyond cognitive outcomes to include engagement and emotional responses.

## 3 Materials and methods

While traditional physical laboratories play a pivotal role in education, they often require significant infrastructure and resources. In contrast, visuo-haptic environments can offer a more cost-effective and accessible alternative for simulating physical experiments, thereby making advanced learning experiences more widely available. In this context, the study focused on developing three distinct visuo-haptic simulators to improve the teaching of classical physics concepts through interactive engagement. The selected scenarios were selected for their potential to benefit significantly from haptic feedback: (a) a block resting on a flat surface with friction; (b) equilibrium of forces (balance); and (c) equilibrium of forces (tug-of-war). These scenarios were created using the HaDIU (Haptic Device Integration for Unity) framework (Escobar-Castillejos et al., 2020). This plugin is accessible via the following link. The force feedback in these simulators was provided through a Novint Falcon haptic device, chosen for its affordability and its capability to deliver three degrees of freedom (DOF) for both spatial input and haptic output. The integration of the Novint Falcon with the Unity3D engine, via the HaDIU plugin, enabled a seamless experience of graphical and haptic renderings.

Each simulation incorporated Unity’s graphical user interface (GUI), which allowed users to control and visualize essential parameters for each scenario, facilitating a hands-on learning experience. The GUI was designed to be intuitive, ensuring that users could easily interact with the simulation and receive immediate haptic feedback relevant to the physics concepts being explored. This approach aimed to not only make the learning process more engaging but also to provide insights into the usability and educational value of visuo-haptic simulations in teaching complex physics concepts. In the following subsections, a detailed description of each scenario is provided.

### 3.1 Design of the visuo-haptic simulators

#### 3.1.1 Block resting on a flat surface with friction

**FIGURE 1:**
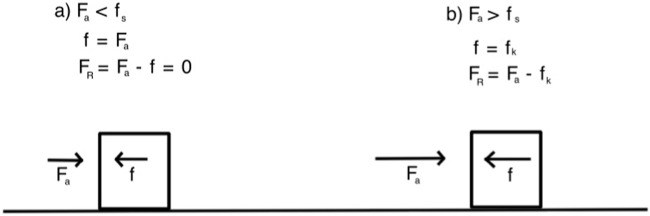
Force diagram for a block resting on a flat surface with friction.

This scenario aimed to provide students with the opportunity to observe and understand the concept of frictional force *f* by simulating a block sliding on a flat surface when a force is applied to it (Figure 1)

Let $\vec{F_{a}}$ be the force applied by the user in a direction orthogonal to the box’s surface, and *F*_*a*_ its magnitude. Similarly, $\vec{F}$ represents the force detected by the simulator’s sensors, and *F* is its magnitude. *μ*_*s*_ corresponds to the static friction coefficient, and *μ*_*k*_ to the kinetic friction coefficient, where it always holds that *μ*_*s*_ > *μ*_*k*_. The magnitude of the friction force is considered as *f*, with *f*_*s*_ representing the static friction force magnitude and *f*_*k*_ the kinetic friction force magnitude. Additionally, *m* corresponds to the simulated mass value, and *P* is the magnitude of the box’s weight. Starting from the initial state of rest, the resultant force $\vec{F_{R}}$ can be determined as follows:

If *F*_*a*_ < *f*_*s*_ = *μ*_*s*_ *P*, then $\vec{F}=-\vec{F_{a}}\Rightarrow \vec{F_{R}}=0$ .

If *F*_*a*_ ≥ *f*_*s*_ then $F=f=f_{k}=\mu_{k}P\Rightarrow \vec{F_{R}}=\left(F_{a}-f_{k}\right)\hat{ı}$ .

Where $\hat{ı}$ represents the unit vector on the horizontal axis.

**FIGURE 2:**
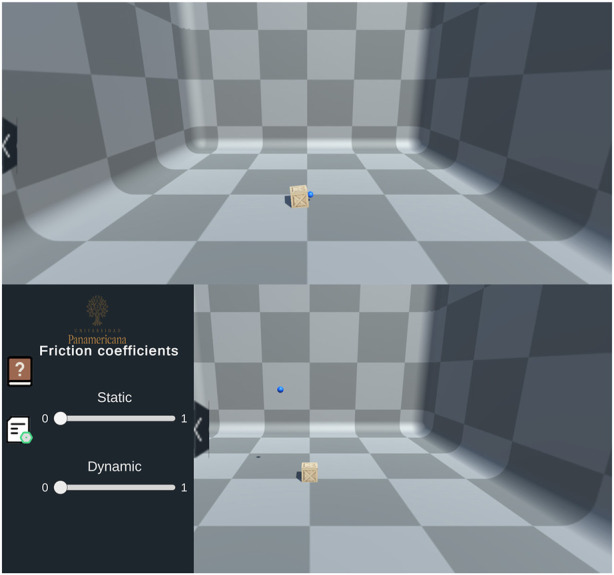
“Block resting on a flat surface with friction” Scenario: (Top) Initial scenario setup; (Bottom) Simulation Parameters.

Consequently, users could apply a force (*F*_*a*_) to the block by using the haptic interaction point (HIP), which interacts with and penetrates the virtual object to simulate the applied force. On the other hand, the ideal haptic interaction point (IHIP) acts as a visual representation, depicting the handle of the haptic device and illustrating the contact point of the HIP within the virtual environment, as demonstrated in Figure 2. Users could grasp the block, allowing them to perceive the virtual weight of the block. The haptic device transmitted the *F*_*R*_, whether it was in a static or kinetic state, to the user using the *F*_*a*_ value.

#### 3.1.2 Equilibrium of forces (balance)

**FIGURE 3:**
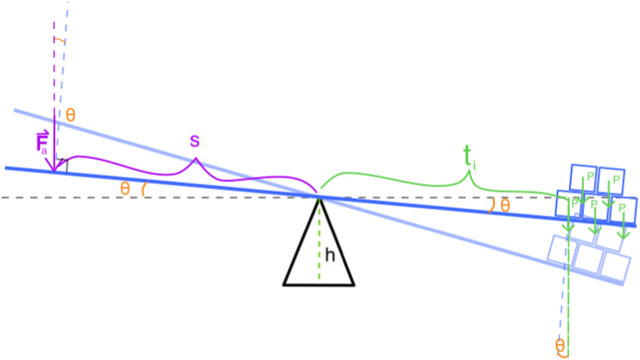
Force diagram for the balance simulator.

The aim of this scenario was for students to interact with the system to understand the concept of torque in terms of the magnitude of the applied forces and their points of application. To determine the resultant force in the simulation, the following considerations, as seen in Figure 3, were taken into account:

The center of rotation is situated at the origin of a Cartesian plane. The variable *L* denotes the distance from the center of rotation to each end of the table. The vector $\vec{F_{a}}$ represents the force that the student applies vertically downward on the left side of the table, and *F*_*a*_ is the magnitude of this force. The term *s* indicates the distance from the center of rotation to the point on the left side where the force $\vec{F_{a}}$ is applied. Conversely, on the right side, the centers of mass for each of the boxes *i* (where *i* ∈ 1, 2, 3, 4, 5) are positioned at distances *t*_*i*_. It is assumed that all boxes have an identical weight, denoted as *P*. The variable *h* represents the height of the center of rotation above the base, while *θ* is the angle that the table makes with the horizontal. The force necessary to maintain the system in equilibrium is described by the vector $\vec{F}$ , with *F* being its magnitude. Consequently, *F*_*R*_ is defined as the resultant force acting on the system.

To obtain the resultant force, it is necessary to know in advance the force $\vec{F}$ that the simulator will produce. This force will be of the same magnitude as what the student would need to apply to maintain the simulation in equilibrium. Starting from the condition of rotational equilibrium, we have:

∑τ=0

$$\Rightarrow \;Fs\;cos\left(\theta \right)=\overset{n}{\underset{i=1}{\sum }}Pt_{i}\;cos\left(\theta \right)$$

Where *n* is the number of boxes in the simulation. Since *P* and cos(*θ*) do not depend on *i*, they can be factored out of the sum. In this way, terms dependent on the angle are canceled on both sides of the equation.

$$Fs=P\overset{n}{\underset{i=1}{\sum }}t_{i}\;\Rightarrow F=\frac{P}{s}\overset{n}{\underset{i=1}{\sum }}t_{i}\;$$

Therefore, for a given number of boxes *n*, the force on the sensor will depend solely on the position *s* and the lengths *t*_*i*_.

$$F\left(s,t_{1},\ldots ,t_{n}\right)=\frac{P}{s}\overset{n}{\underset{i=1}{\sum }}t_{i}$$

Thus, the resultant force on the systems will be:

$$\vec{F_{R}}=\vec{F_{a}}+\vec{F}\left(s,t_{1},\ldots ,t_{n}\right)$$

Since $\vec{F_{a}}$ is applied downward on the left side of the pivot point:

$$\vec{F_{R}}=\left(\frac{P}{s}\overset{n}{\underset{i=1}{\sum }}t_{i}-F_{a}\right)\hat{ȷ}$$

Where $\hat{ȷ}$ represents the unit vector on the vertical axis.

**FIGURE 4:**
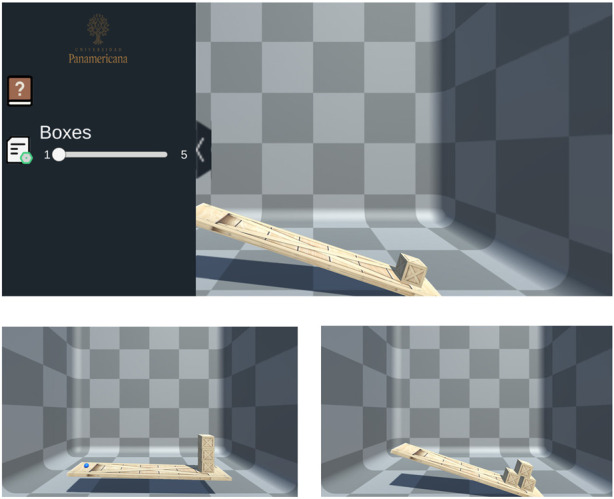
*“*Equilibrium of forces (balance)” Scenario: (Top) Initial scenario setup; (Bottom-Left) Simulation with three stacked boxes vertically; (Bottom-Right) Simulation with three-box pyramid formation.

In this simulation scenario, participants can exert a force, represented as $\vec{F_{a}}$ , through the HIP on the left side of the simulated table (Figure 4). As the user moves the blue ball, which represents the IHIP’s position on this plane, the HaDIU plugin computes the resultant force $\vec{F_{R}}$ , by using the HIP’s position combined with Unity’s physics engine values to provide appropriate haptic feedback to the user. Additionally, users can move the boxes on the right side of the table freely by approaching them with the IHIP. Upon contact with a box, pressing the button on the haptic device allows the user to ‘grab’ and reposition the box. Modifying the position of the boxes changes the distribution of forces and the resultant torque in the calculation, enabling students to physically sense and understand the changes in equilibrium.

#### 3.1.3 Equilibrium of forces (tug-of-war)

**FIGURE 5:**
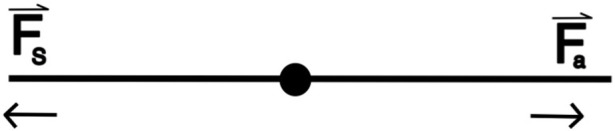
Force diagram of equilibrium of forces (tug-of-war).

This scenario was designed to help students understand the concept of equilibrium concerning the magnitude of applied forces through a simulation that represents accelerated motion on the rope, which depends on the resultant force applied to it (Figure 5).

Let *F*_*s*_ be the force initially exerted on the rope by the simulator, and *F*_*a*_ be the force applied by the student. The resultant force *F*_*R*_ will be:

$$\vec{F_{R}}=\vec{F_{s}}+\vec{F_{a}}$$

**FIGURE 6:**
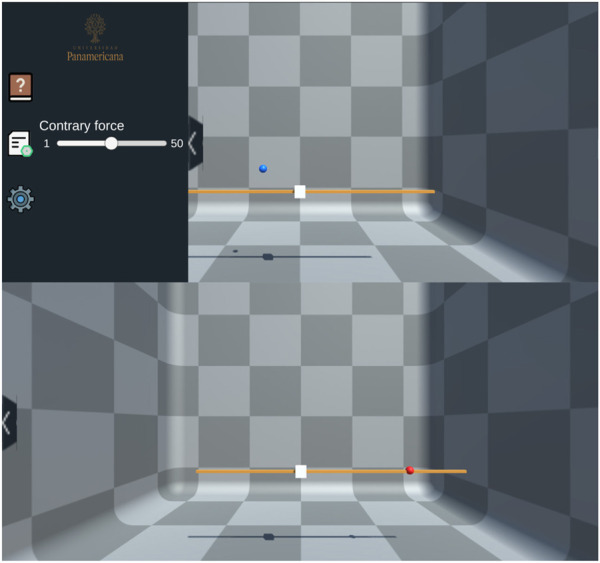
“Equilibrium of forces (tug-of-war)” Scenario: (Top) Initial scenario setup; (Bottom) Simulation with the user applying an opposite force.

In this environment, students were able to test another equilibrium of forces simulator (Figure 6). Through the HIP, participants could exert an external force, represented as *F*_*a*_, on the virtual rope. The blue and red spheres represent the same IHIP; the blue sphere indicates the HIP’s position when it is not in contact with the rope, whereas the red sphere signifies that the HIP is engaging with the rope. The acceleration and movement of the rope are determined by the interaction between the student’s force and the simulator’s preset force *F*_*s*_. It should be noted that although users can move freely within the simulation space, the HIP’s movement is constrained to the *x*-axis once contact is made with the rope. This design constraint was considered to simulate the feeling of the opposing force, thereby enriching the user’s haptic experience and providing a more realistic sense of equilibrium.

### 3.2 Measures

**FIGURE 7:**
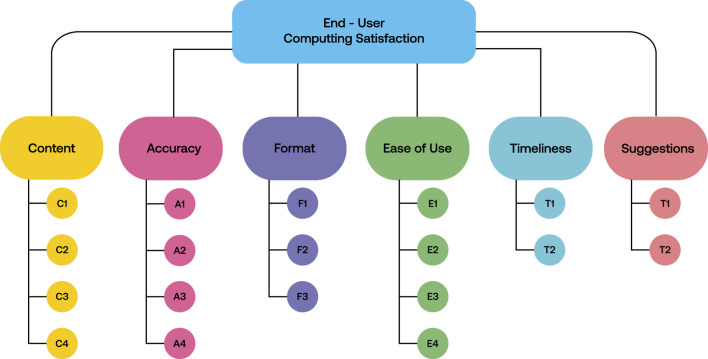
Proposed end-user computer satisfaction survey. C1: Do the simulators provide the precise information you need to understand the activities? C2: Do the simulators’ configuration parameters meet your needs? C3: Do the simulators provide output values that seem to be helpful in your activity? C4: Do the simulators provide sufficient instructions to perform activities? C5: Do you consider the simulators could support you in classes? A1: Are the output values accurate? A2: Do you feel the kinesthetic perception is realistic? A3: Do you feel the visual aspects are realistic? A4: Do you find the simulators dependable? F1: Do you think the graphical user interface (GUI) presents information in a useful format? F2: Is the information clear? F3: Do you find the visualization of the visuo-haptic simulators attractive? E1: Are the simulators user-friendly? E2: Are the simulators easy to use? E3: Did you feel motivated when you were using the simulators? E4: Did you feel the information offered by the simulators was useful? T1: Do you get the simulators’ information you need in time? T2: Do the simulators provide real-time rendering? S1: What suggestions do you have to improve the simulators to better support you in your learning process? S2: General comments.

To make it suitable for multi-modal simulations, Doll and Torkzadeh’s end-user computer satisfaction survey (Doll and Torkzadeh, 1988) was modified (Figure 7). The modified survey included questions that spanned various aspects of the simulators, addressing clarity, output value accuracy, visual and kinesthetic realism, reliability, user interface efficacy, understandability, motivation, and real-time information delivery. These questions were structured around a five-point Likert scale, with five representing the highest level of satisfaction or agreement and 1 being the lowest. However, items S1 and S2 were conceived as open-ended questions, allowing participants to provide suggestions and general comments. The survey was delivered via Google Forms, and the data collected was analyzed using Python with the aid of the Pandas and Matplotlib libraries.

### 3.3 Participants

During the academic semester of August to December 2023, an experimental group of 45 engineering students from Universidad Panamericana, ranging from the 1st to the 7th semester and with differing levels of familiarity with the topics, participated in testing sessions for the three simulators developed. These sessions, spanning 1 week, aimed to assess the usability of the visuo-haptic simulators and gather students’ perceptions of their utility in enhancing the learning experience of classical physics concepts.

Each session started with a detailed introduction to the simulators, presenting their features and the tasks participants were expected to complete. Positioned in front of computers equipped with the simulators and the Novint Falcon haptic device, students were able to adjust the device for their dominant hand, ensuring comfort and ease of use. Through interaction with the simulators using the Novint Falcon, participants gained an intuitive understanding of the forces associated with the presented classical physics concepts. Through the graphical user interface (GUI), participants use the mouse to modify simulation parameters, including the number of boxes, static and dynamic friction coefficients, and opposing forces. The GUI was intentionally designed to reduce the need for users to alternate between the mouse and the Falcon haptic device, ensuring a seamless and intuitive interaction. Most parameter adjustments are completed before engaging with the Falcon, allowing for uninterrupted kinesthetic feedback during the simulation. After adjustments, participants were asked to access the ‘Results Tab’ to monitor the simulation’s outcomes. This strategy guarantees that participants can both visually and kinestheticly perceive the immediate effects of their modifications. By minimizing device switching, this approach not only preserves the ergonomic integrity of the setup but also sustains an immersive and efficient simulation environment.

The average exploration time was approximately 30 min per student, focusing on engaging with the simulations rather than a quantitative measure of learning outcomes. Following each session, feedback was collected using the adapted end-user computing satisfaction questionnaire. This feedback was used to measure the simulators’ effectiveness in providing an intuitive and engaging learning experience. This approach allowed the study to examine the educational potential of visuo-haptic technology from the perspective of user satisfaction and perceived learning enhancement.

## 4 Results

**FIGURE 8:**
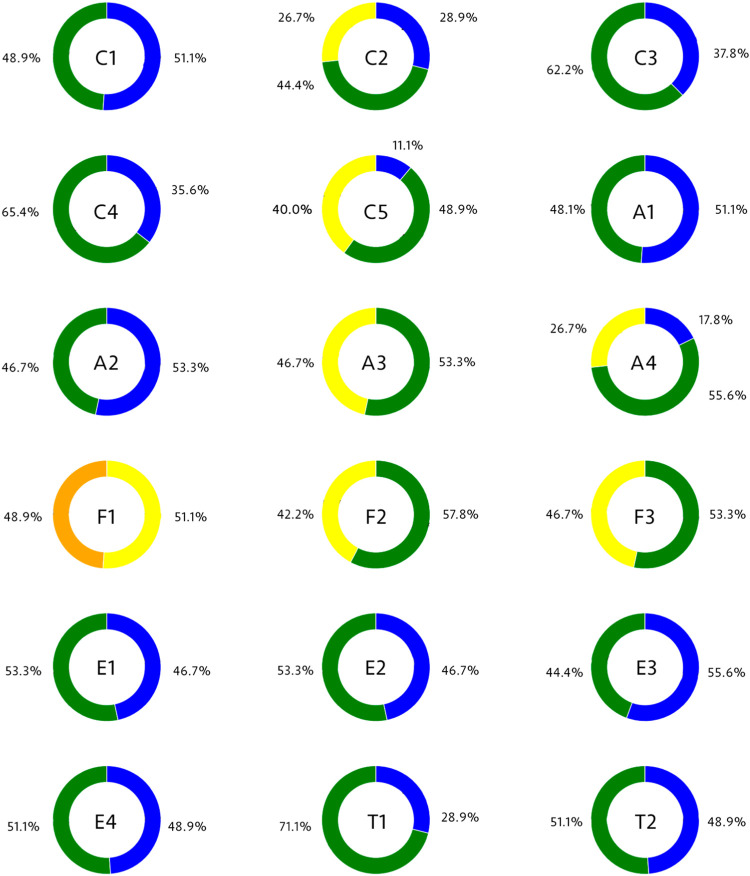
Obtained results in the proposed end-user computer satisfaction survey. The colors in the pie. charts correspond to values on a 5-point Likert scale: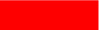 Represents the lowest value.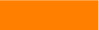 Represents the 2nd lowest value.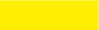 Indicates a midpoint or neutral response.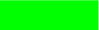 Represents the 4th value, leaning towards the positive end of the scale.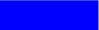 Represents the highest or most positive value.

The satisfaction survey results, as depicted in Figure 8, provide insights into participants’ perceptions of the visuo-haptic technology’s effectiveness in enhancing the learning experience.

### 4.1 Content (C1-C5) ratings distribution

Analysis of the content quality ratings reveals that most responses range between 4 and 5, suggesting that participants generally perceived the visuo-haptic technology’s effectiveness in providing an engaging learning experience. However, the variance in ratings for C2, concerning specific content-related aspects of the simulators, suggests room for improvement in content delivery. Specifically, feedback on C2 highlighted a need for clearer explanations of complex physics concepts and suggested incorporating more varied learning materials to cater to different learning styles. For example, participants expressed a desire for more interactive elements, such as quizzes or real-time feedback, to test their understanding throughout the simulation. This feedback is instrumental in guiding enhancements to the simulator design, ensuring content is both accurate and presented in a manner that is accessible to learners with diverse preferences.

### 4.2 Accuracy (A1-A4) ratings distribution

Ratings on the accuracy of the simulations validate the simulators’ potential to provide a realistic representation of physics principles, with A1 and A2 indicating strong user approval. However, the varied responses for A3 and A4 suggest opportunities for further research into how these elements influence the overall educational value of the simulations. Such insights could guide enhancements in the accuracy and realism of simulated physics phenomena.

### 4.3 Format (F1-F3) ratings distribution

The distribution of ratings for the presentation format reveals areas for improvement, particularly for F1, which received lower scores. This feedback points to a possible misalignment between the simulators’ educational content and its presentation. Enhancing the format could significantly impact user engagement and learning experiences. Conversely, the positive responses to F2 and F3 suggest that some aspects of the format are effective and should be retained or further developed in future developments.

### 4.4 Ease of use (E1-E4) ratings distribution

The ease of use ratings were consistently positive, which highlights the simulators’ user-friendly design and underscores their potential as valuable educational tools in physics learning. These results suggest that visuo-haptic simulators, with further development, could become integral for teaching complex concepts that benefit from kinesthetic learning experiences.

### 4.5 Timeliness (T1-T2) ratings distribution

The timeliness ratings, especially the inclination towards a score of five for T2, demonstrate participants’ satisfaction with the simulators’ responsiveness and promptness. This feedback highlights the potential of visuo-haptic environments to deliver immediate, interactive feedback, a key factor in facilitating an effective and engaging learning environment.

### 4.6 Box plot for distribution of responses to each question

**FIGURE 9:**
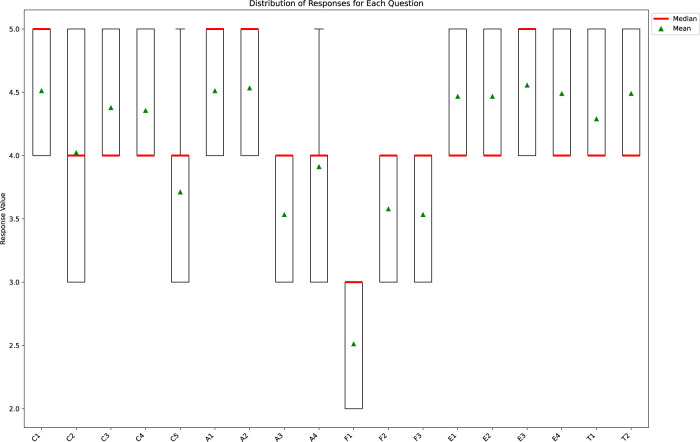
Box plot for distribution of responses to each question.

Box plots provide a visual representation of the central tendency, variability, and presence of outliers in the distribution of responses for each question (Figure 9). For example, the interquartile range (IQR), which is defined as the difference between the first quartile (the lower boundary of the box) and the third quartile (the upper boundary of the box), provides insight into the central 50% of the data distribution for each question.

Based on the data, the box plot analysis offered a clear depiction of the user feedback, capturing both general trends and the range of responses. While the simulators were generally well-received, indicated by a predominance of ratings in the 4 to five range, some variations and outliers suggest some aspects did not fully meet all participants’ expectations or preferences. These results highlight that while features such as the simulators’ accuracy and ease of use were highly rated, the presentation and format, particularly for F1, require further attention to meet user satisfaction across all dimensions of the simulator experience.

## 5 Discussion

The satisfaction survey results indicated a generally positive perception of the visuo-haptic technology, with content and accuracy ratings being particularly strong. However, areas for improvement were identified in the content delivery specifics and presentation format.

### 5.1 Comments obtained in the suggestions category

The qualitative feedback received from participants shows the positive impact of the visuo-haptic simulators on the learning experience. Among the 33 participants who provided comments, a significant majority (63.6%) praised the simulators for providing a more immersive understanding of physics concepts than traditional methods. Common feedback included appreciations such as ‘they facilitated a deeper comprehension of classical concepts’ and ‘the simulations were engaging and instructive,’ underscoring the value of incorporating interactive, multi-modal tools in educational settings.

These results not only highlight the potential of visuo-haptic technologies to enhance conceptual understanding beyond traditional methods but also point to specific areas for enhancement. Participants also recommended expanding the simulations’ content to ‘cover a wider range of physics topics’ and to ‘improve the visual interface for more intuitive 3D navigation.’ Furthermore, the suggestion to ‘integrate learning features’, such as quizzes, underlines a strong interest in personalizing the learning experience to support diverse learning styles and speeds.

### 5.2 Comparison with research questions

This study contributes to understanding the potential of visuo-haptic technology in enhancing the educational experience, particularly in how it influences students’ engagement with and perceptions of physics concepts. However, it also highlights the need for a more in-depth analysis of how interactions with visuo-haptic simulators affect long-term knowledge retention and fit diverse learning preferences.

According to the results, multi-modal technology significantly influences students’ engagement and their understanding of basic physics concepts, thereby answering our initial research question. Feedback and rating distribution indicate that multi-modal technologies improve student engagement and enrich their comprehension of physics topics. Positive feedback indicates that these tools make abstract topics more understandable and clear, improving learners’ involvement and understanding.

Responses about the user-friendliness and educational value of visuo-haptic simulators provide clear answers to our second question. The consistently high scores for simplicity of use emphasize the user-friendly design of the simulators. Simultaneously, content evaluations and feedback show the substantial instructional value of the simulations. Recommendations to expand the content and incorporate diverse learning materials indicate ways to enhance their educational impact.

Regarding our third question, the diverse feedback, provided by the different metrics along with the qualitative comments, indicates specific areas for improvement, such as content delivery and presentation format. This feedback highlights the importance of interactive, multi-modal educational tools and provides direction for future improvements. Recommendations for more interactive features and expanding the variety of topics are crucial to guide the ongoing development of multi-modal educational technology.

The conducted analysis underscores the simulators’ positive reception, aligning with our research questions regarding their usability and intuitive design. Nonetheless, the presence of ratings in the 3-4 range indicates areas requiring attention. While the simulators’ accuracy and ease of use were praised, improvements in the format, especially concerning F1, are necessary to elevate the overall learning experience. This aspect suggests the importance of developing educational tools that are not only pedagogically effective but also broadly engaging and accessible.

### 5.3 Comparison with related research

This study extends existing research in the field by investigating not only the direct educational effects of visuo-haptic simulators but also user satisfaction and engagement. Walsh and Magana’s study of physical manipulative tools and visuo-haptic simulations for teaching statics supports this approach. They focused on how different kinds of visual and haptic feedback can help students learn about friction Walsh and Magana (2023).

This research investigates the precise effects of multi-modal simulation on student engagement and comprehension. It is based on the growing consensus in the educational technology area that active learner engagement with kinesthetic interaction is essential for improving conceptual understanding and improving outcomes in STEM education. Additionally, comparing the obtained results with related research indicates a consistent finding that interactive technologies can significantly enhance learning experiences. However, the emphasis on user feedback for specific improvements provides unique insights into optimizing visuo-haptic technologies for educational purposes. By integrating these insights with quantitative data, we gained a comprehensive understanding of user experiences and preferences. This feedback provides a solid foundation for future development in multi-modal educational technology. With a focus on user-centric design and educational effectiveness, future simulators could be refined to better meet the diverse needs of learners. This approach promises not only to enhance engagement but also to deepen the understanding of complex physics concepts.

### 5.4 Limitations and recommendations for further studies or analyses

Lastly, the authors acknowledge that a limitation of this study is its sole reliance on subjective feedback, which offers a limited view of the simulators’ educational impact. Incorporation of objective measures, such as engagement metrics and performance assessments, would offer a more comprehensive assessment of the simulators’ learning impact and efficacy. Future developments of new visuo-haptic environments will aim to integrate objective metrics of learning outcomes to ensure a thorough evaluation of the simulator’s qualitative and quantitative advantages. Additionally, further development should consider feedback on content delivery and presentation format to enhance the educational impact of visuo-haptic technologies.

## 6 Conclusion and future work

In this study, we developed three visuo-haptic simulators to investigate the potential benefits of multi-modal learning experiences within STEM education. We focused on three classical physics concepts: friction on a flat surface, balance in force equilibrium, and tug-of-war dynamics. A graphical user interface was integrated to facilitate user interaction, providing instructions, allowing for the adjustment of simulation parameters, and displaying force values as a direct feedback mechanism.

Moreover, an enhanced survey was utilized to measure end-user computing satisfaction. It was adapted to capture feedback on the multi-modal simulation experience. This study presented an alternative methodology that could potentially enable more structured evaluations in this area.

A detailed study involving students was conducted to determine the level of understanding and the insights obtained through interactions with multi-modal simulators. Results suggest that using haptic devices along with carefully planned visuo-haptic ecosystems could help participants understand the complex nature of forces in classical physics and where they come from. The feedback obtained regarding the simulations was predominantly good. Feedback collected through the enhanced survey highlighted the simulators’ positive impact on participants’ understanding and engagement with physics concepts.

Despite the overall positive reception, an analysis of open-ended responses revealed areas for further improvement. Key themes from this analysis include:

1. Participants expressed a desire for simulations covering a broader spectrum of physics phenomena, suggesting the need for expanding our simulator library to encompass more varied and complex concepts.
2. Enhancements to the visual representation, during the movement of the haptic device avatar for more intuitive navigation and interaction were frequently requested, indicating a priority for future iterations to focus on streamlining the user experience.
3. The call for integrating quizzes and additional educational resources highlights a demand for personalized learning paths within the simulators, catering to diverse educational backgrounds and learning speeds.

Future research will focus on these insights, starting with a comparative analysis aimed at quantifying the educational impact of visuo-haptic simulators against traditional teaching methods. Scheduled for January-May 2024, this study will adopt a rigorous methodology designed in collaboration with faculty members, ensuring a comprehensive evaluation of learning outcomes. Additionally, recognizing the vast potential of classical physics for educational exploration, we plan to convene focus group discussions to strategically select topics for subsequent simulator development. These discussions will prioritize areas where visuo-haptic integration offers maximal educational value, thus refining our approach to enhancing engagement and comprehension of complex physics concepts. Through this targeted future work, we aim to address the identified areas for improvement, further advancing the field of multi-modal educational technology and enriching the learning experience for students.

## Data Availability

The original contributions presented in the study are included in the article/Supplementary material, further inquiries can be directed to the corresponding author.
